# Integration of Riboflavin-Modified Carbon Fiber Mesh Electrode Systems in a 3D-Printed Catheter Hub

**DOI:** 10.3390/mi15010079

**Published:** 2023-12-30

**Authors:** Charnete Casimero, Robert B. Smith, James Davis

**Affiliations:** 1 School of Engineering, Ulster University, Belfast BT15 1ED, UK; casimero-c@ulster.ac.uk; 2 Institute for Materials and Investigative Sciences, University of Central Lancashire, Preston PR1 2HE, UK; rbsmith@uclan.ac.uk

**Keywords:** catheter, CLABSI, infection, sensor, screen-printed electrode, SPE, riboflavin, total parenteral nutrition, TPN

## Abstract

Background: Catheter line infection is a common complication within clinical environments, and there is a pressing need for technological options to aid in reducing the possibility of sepsis. The early identification of contamination could be pivotal in reducing cases and improving outcomes. Method: A sensing rationale based on a riboflavin-modified electrode system integrated within a modified 3D-printed catheter needle-free connector is proposed, which can monitor changes in pH brought about by bacterial contamination. Results: Riboflavin, vitamin B2, is a biocompatible chemical that possesses a redox-active flavin core that is pH dependent. The oxidation peak potential of the adsorbed riboflavin responds linearly to changes in pH with a near-Nernstian behavior of 63 mV/pH unit and is capable of accurately monitoring the pH of an authentic IV infusate. Conclusions: The proof of principle is demonstrated with an electrode-printed hub design offering a valuable foundation from which to explore bacterial interactions within the catheter lumen with the potential of providing an early warning of contamination.

## 1. Introduction

The insertion of an intravascular catheter is commonplace within hospital environments, with 30–80% of patients receiving a peripheral device upon admission. These are used for a range of clinical procedures that include providing fluids, nutrition, and antibiotics, as well as enabling more complex treatments such as chemotherapy and dialysis [1]. It has been estimated that some 300 million peripheral intravascular catheters (PIVCs) are inserted annually in the US, along with 3–5 million central venous catheters (CVCs) [1,2]. The former is the standard access device positioned on the hand or forearm and used for short-term interventions (typically several days at a time), whereas the latter is retained for much longer periods (months to years) [1,2,3]. As can be expected with any device penetrating the skin and accessing the venous network, infection is a constant hazard with increased duration over which CVC devices are placed and has long been recognized as particularly problematic [1,2,3,4]. The introduction of patient–clinician care bundles, greater adherence to aseptic techniques and monitoring protocols, and the introduction of lock solutions have been shown to significantly reduce catheter line-associated bloodstream infection (CLABSI) rates [5,6,7]. This is evidenced by a sustained reduction in annual CLABSI incidents recorded by the US Centers for Disease Control and Protection (CDC) [8]. Yet, despite such improvements, infection rates remain far from acceptable and present a problem for countries irrespective of income or development status [9]. The aim of the present communication is to explore the design and implementation of an electrochemical sensor that could be used to measure pH within the access line and, therein, offer the possibility of indirectly detecting the presence of microbial growth.

The development of needle-free hub connectors (NFCs) was originally spurred by the need to reduce the risk of needlestick injuries and the possibility of incurring the transmission of blood-borne diseases [10]. NFCs terminate the user end of the CVC line and enable a facile route for intravenous access either to or from the body [11]. Although these can be considered the first line of defense against catheter contamination, numerous investigations have subsequently shown that NFCs are, in fact, one of the more common sources of infection [4,11,12,13,14]. The presence of dead space between the outward septum seal and the body of the NFC in many designs can offer an opportunity for microorganism colonization and biofilm formation. As such, these areas are ideally positioned to lead to catheter-line-associated bloodstream infection. While the adoption of rigorous aseptic techniques and “scrubbing the hub” are advocated as a means of reducing infection risk [14,15,16], contamination remains an ever-present issue. The adherence of the healthcare practitioner (or patient) to such principles, however, can be a limiting factor.

**Figure 1:**
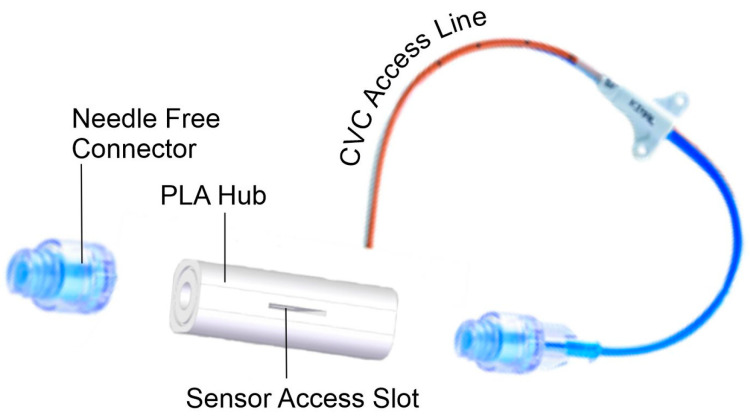
Integration of disposable electrode assemblies within a conventional intra-venous-access catheter.

The regular replacement of the NFC is one approach to mitigating the build-up of the biofilm [14], but the integration of sensors within the core design could enable the early identification of contamination. However, the potential advantages could be far-reaching where data analytics are employed to identify frequent contamination that could pinpoint poor IV-line management (either by the patient or healthcare provider). This could be invaluable in optimizing the aseptic technique within a clinical unit and thereby reducing infection through proactive prevention [14]. The challenge, however, rests with providing sensors that are sufficiently small, low-cost, and readily integrated with NFC designs. The approach taken here was to employ a 3D-printed hub extension incorporating microporous carbon fiber electrodes, as indicated in Figure 1. In this case, the hub unit was separated from the NFC in order to facilitate the characterization of the sensors, but it could be envisaged that, ultimately, the sensors would be integrated directly within the NFC itself.

The adoption of carbon fiber-based systems provides a low-cost option, with the wider operational mode expected to be similar to that observed with home glucose meters where control electronics are retained, but the sensing strips are discarded after each use. In this case, a NFC hub is typically replaced every 3–4 days of use [17,18]. One possible limitation, however, would be whether or not the carbon fiber mesh electrode possesses the electrochemical characteristics necessary for facilitating the unambiguous identification of pH. The adoption of carbon fiber as the foundation of an electrochemical sensor has been widely used for sensing applications—typically for the detection of neurotransmitters [19,20,21] and other biologically relevant agents [22,23,24,25]. In this case, carbon fiber electrodes were functionalized with riboflavin, and the pH-sensitive nature of its electrochemical properties was harnessed as an indirect means of measuring pH [26,27,28].

**Figure 2:**
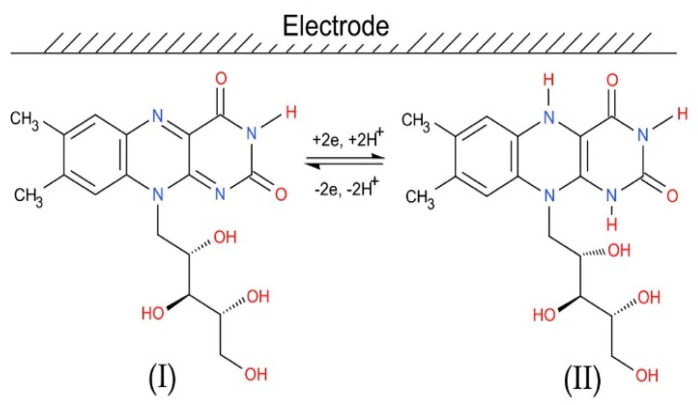
Electrochemical redox transitions of riboflavin at an electrode detailing the reduction (I → II) and corresponding oxidation (II → I).

The redox transitions of riboflavin are highlighted in Figure 2, where the reduction (I → II) and oxidation (II → I) processes are based on two-electron/two-proton transfer [26,27,28]. It was envisaged that an adsorbed layer of riboflavin could enable the pH to be determined through the indirect voltammetric measurement of the oxidation peak position, and its applicability as a pH-sensitive probe has been previously demonstrated through its use with a laser-induced graphene sensor [28]. Its application here is particularly pertinent given its inherent biocompatibility and, thus, should it leach into the solution, its presence within the access line would not pose any clinical complications. It should be noted that riboflavin is a key component of nutrition solutions delivered via IV, which are at a far greater concentration than that used here, where the riboflavin is present only as an adsorbed layer on the carbon fiber electrode.

The integration of carbon fiber mesh electrodes with the hub-based design highlighted in Figure 1 has been investigated, and the electrochemical properties of the resulting sensors are characterized. The ability of this system to monitor the pH on a periodic basis, in alignment with the normal operation of the needle-free connector, was evaluated through the analysis of an authentic total parenteral nutrition infusate.

## 2. Materials and Methods

**Figure 3:**
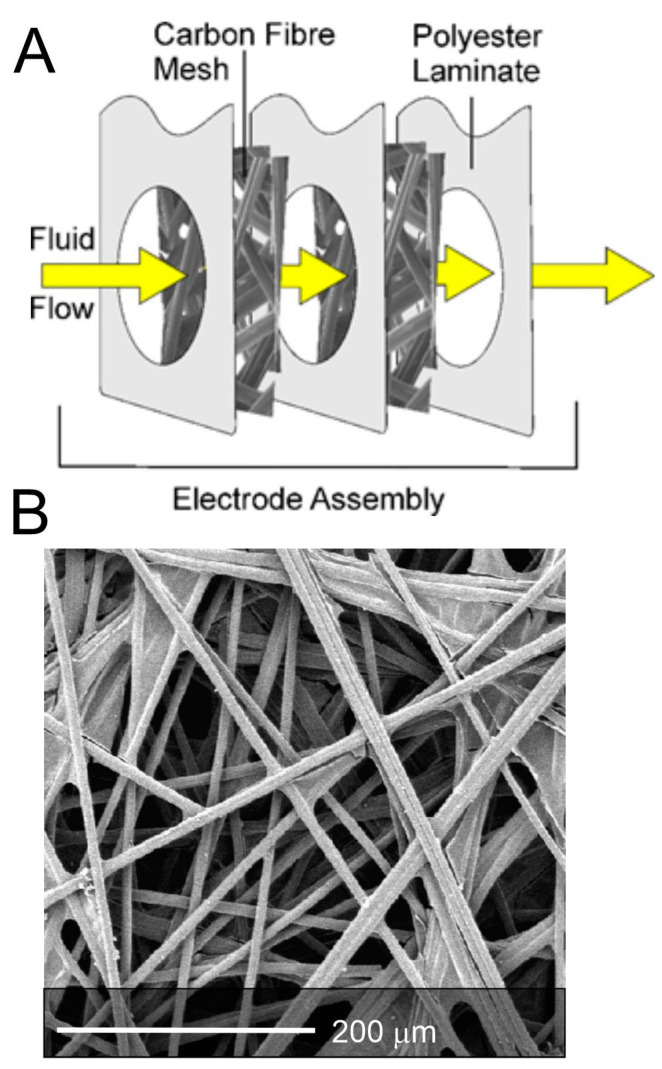
(**A**) Carbon fiber electrode configuration and (**B**) scanning electron image of the Toray carbon fiber mesh.

Chemicals were obtained from Sigma Aldrich (Gillingham UK) and were the highest grade available, used without further purification. Toray carbon fiber (TGP-H-60, 19 × 19 cm) was used as the electrode substrate and was purchased from Alfa Aesar (Thermo Fisher Scientific, Altrincham UK). Britton Robinson buffers (equimolar 0.04 M acetate, phosphate, and borate containing 0.1 M KCl) were used throughout. Electrochemical analysis was conducted at 22 ± 2 °C using a µAutolab Type III potentiostat. Initial investigations employed a standard three-electrode configuration with a two-electrode system employed for the modified NFC. Carbon fiber served as the working electrode and was modified through electrochemical anodization (+1.5 V, 300 s, 0.1 M NaOH) and then functionalized with riboflavin (physio-sorption) by simply placing the electrode within a riboflavin solution (250 µM, pH 7). The three-electrode configuration was completed by employing platinum wire and a conventional Ag|AgCl half cell (3M KCl, BAS Technicol UK) as the counter and reference electrodes, respectively. The 3D-printed NFC hub integrated with carbon fiber electrodes utilized a two-electrode system. The carbon mesh was heat-sealed within a pre-patterned polyester laminate. In this case, an 8 × 4.5 mm window exposes the active carbon fiber mesh using a method similar to that described previously [27,28]. A schematic of the basic electrode configuration is indicated in Figure 3A. While one electrode served as the working electrode (modified with adsorbed riboflavin), the combined counter-reference electrode comprised electrodeposited g, which was subsequently chloridized to form a Ag|AgCl pseudo reference. The deposition of silver was achieved by employing chronoamperometry (−0.1 V for 300 s, 10 mM AgNO_3_/0.1 M HNO_3_). The electrodes were then chlorodized using a single cyclic voltametric sweep (−1.0 V → 0.6 V → −1.0 V) in 0.1 M of a KCl electrolyte. The nature of the carbon fiber network is highlighted in the scanning electron micrograph detailed in Figure 3B, and it is clear that, when inserted within the hub systems, the microporous framework enabled fluid flow without any appreciable back pressure.

### Electrode—Printed Hub Design

**Figure 4:**
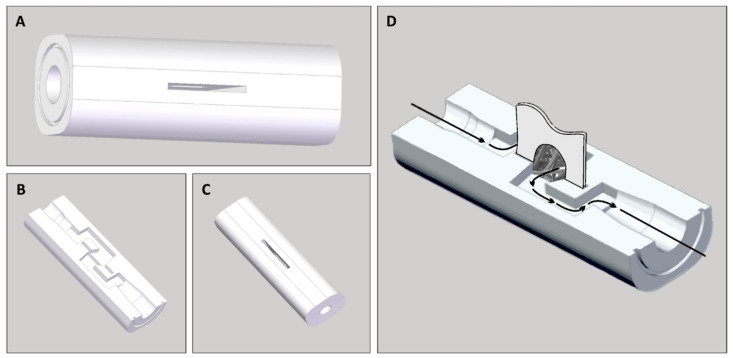
Schematics highlighting the external (**A**,**C**) and internal (**B**) design of the needle-free hub and the proposed flow path (**D**).

The modified hub was printed using an Ultimaker 2+ fused deposition modeling printer (0.4 mm nozzle) with a 3 mm polylactic acid (PLA) filament and was printed in a single step. The initial NFC hub system is presented in Figure 4, where it can be envisaged how the 3D-printed hub is readily added to existing CVC lines. Rather than a straight channel, the design was later enhanced (Figure 4B) so that the solution flowed directly through the two-electrode carbon fiber mesh sensor (Figure 4D) inserted into the slot. It was envisaged that any alterations in the solution pH, as a consequence of the action of microorganisms, could be detected and thereby serve to warn either the patient or healthcare practitioner. The transport of fluid within the 3D-printed hub is highlighted in Figure 4D. A core advantage of the 3D-printing approach used here is that the rapid prototyping of the device is relatively simple—allowing fast optimization—and it can be anticipated that its translation to conventional molding practices is straightforward.

## 3. Results

### 3.1. Preliminary Characterization

**Figure 5:**
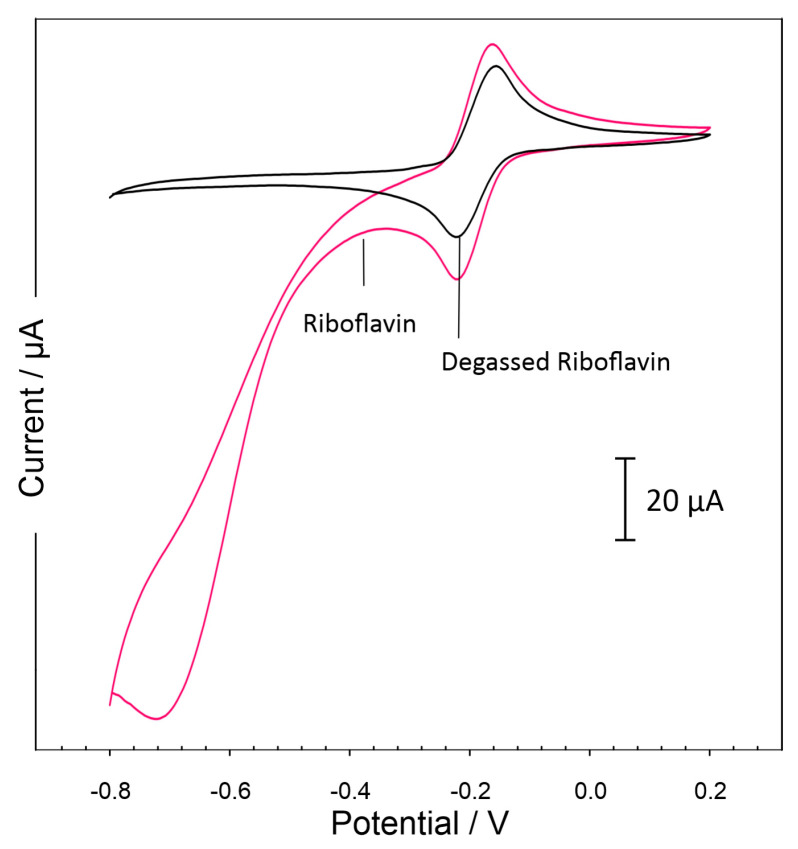
Cyclic voltammetric response of the anodized carbon fiber electrode towards riboflavin (3.19 µM, pH 6.21) before and after degassing with nitrogen. Scan rate: 50 mV/s.

Representative cyclic voltammograms of riboflavin (3.19 µM, pH 6.21) recorded using an anodized carbon fiber electrode (Pt counter, 3 M Ag|AgCl half-cell reference) before and after degassing with nitrogen are compared in Figure 5. Both redox peak processes of riboflavin are well-defined and reside within a moderately cathodic potential region (E°: −0.18 V vs. 3 M Ag|AgCl). In the case where the solution has not been degassed, the reduction of dissolved oxygen can be observed at −0.7 V. This is an irreversible process which, from a diagnostic perspective, could complicate the measurement of the riboflavin reduction peak. As such, the oxidation peak was used as the main diagnostic handle in subsequent studies. This is significant from an initial sensing perspective as it allows the acquisition of riboflavin peak data without undue interference from common electroactive interferences, which are normally oxidized within the anodic region.

**Figure 6:**
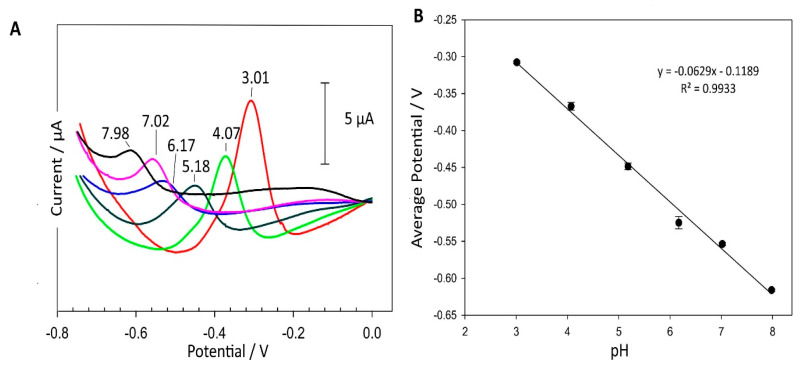
(**A**) Square wave voltammograms detailing the response of a two-electrode riboflavin-modified carbon fiber 3D-printed hub to BR buffers of varying pH. (**B**) Associated regression data. Note: Each point relates to the average of 5 scans and error bars represent the mean standard deviation. Responses were recorded using an integrated solid state (0.1 M KCl) Ag|AgCl pseudo reference.

In previous studies, a modified flavin component was electropolymerized onto the electrode through an oxidative process (typically through the oxidation of a phenol functional group) [27]. Here, the modification of the electrode was achieved through simple physisorption. The immersion of the carbon fiber mesh in a riboflavin solution (250 µM, pH 7) was found to facilitate the adsorption process. This was confirmed through the repetitive rinsing of the electrode with a fresh electrolyte (or buffer) with no perturbation of adsorbed species (assessed via cyclic voltammetry) and with no change in the electrochemical properties from those observed with the electrode’s response to riboflavin in the solution. The effectiveness of the modification procedure on the carbon fiber electrode (Figure 4) was assessed by examining the square wave voltammetric profiles recorded in buffers of varying pH (with no riboflavin in the solution). Square wave voltammetry was initiated at a negative potential of −0.8 V, which was sufficient to induce the immediate reduction in riboflavin and was then swept towards more positive potentials to initiate its re-oxidation (II → I, Figure 2). Square wave voltammetry was selected on the basis of providing the greater resolution of the peak potentials and negating the effects of dissolved oxygen, thereby allowing direct quantitation without the need for degassing. The voltammograms recorded at the two-electrode NFC are detailed in Figure 6A, with the oxidation peak potentials found to shift to more negative potentials with increasing pH. A near-Nernstian behavior was observed with 63 mV/pH unit (E/V = −0.0629 pH—0.1189; N_total_ = 18 (N_pH_ = 6, N_scans/pH_ = 3); R^2^ = 0.9933), which is consistent with the key performance characteristics previously reported with an electropolymerized flavin phenol polymer [27].

**Figure 7:**
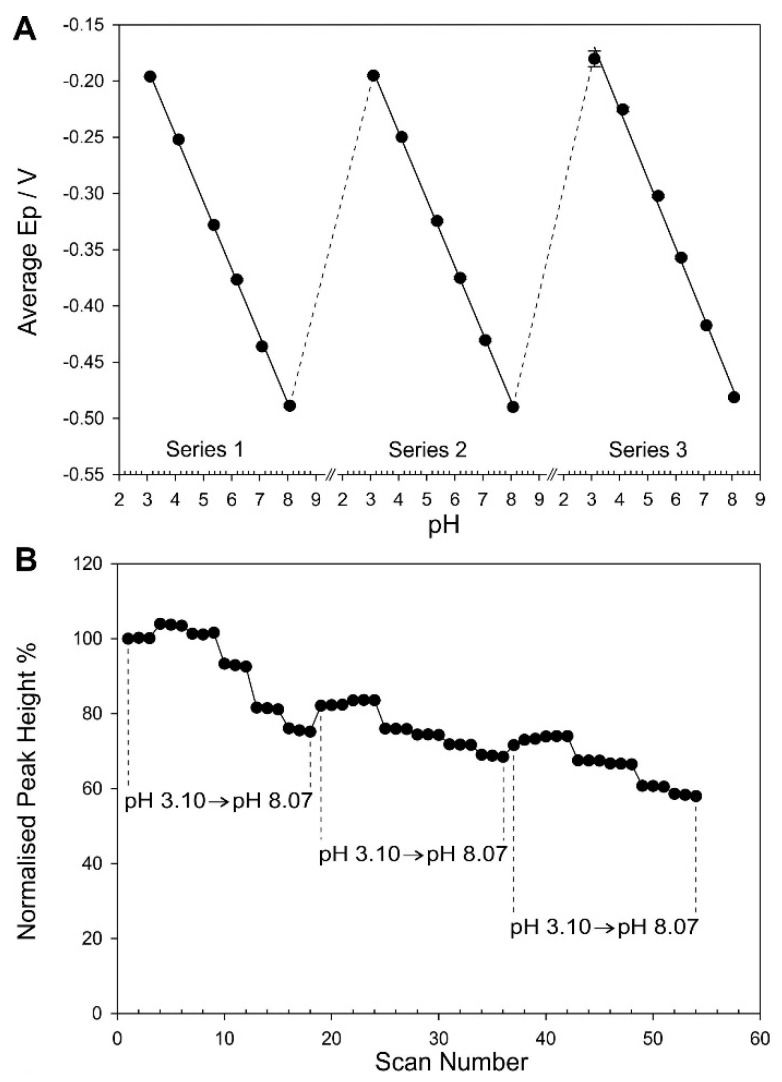
Changes in (**A**) peak potential and (**B**) current magnitude of the physiosorbed riboflavin-modified-anodized carbon fiber electrode as a result of serial scanning in BR buffers of varying pH. Responses were recorded using a 3 M KCl Ag|AgCl half-cell reference.

It has been recommended by the US CDC that NFCs be replaced within 72 h in order to reduce the likelihood of bloodstream infection [14,18]. The sensing-hub approach proposed here must be able to perform repetitive scans throughout a similar period. In order to test the robustness and reversibility of the sensing component, the electrodes (without hub) were cycled through three series of buffer solutions (covering pH 3–pH 8). Variations in the peak potential and peak magnitude during each buffer series are compared in Figure 7. The data in this instance were recorded using a three-electrode system with an external reference (3 M KCl, Ag|AgCl half-cell) to remove any ambiguities associated with the stability of the pseudo-Ag|AgCl reference. It was found that there was a potential drift of 11 mV (0.19 pH unit) over a total of 54 scans. Clearly, a minimal change in the electrode response (potential drift and peak magnitude) are critical consideration where repetitive/periodic monitoring is required. However, it is important to note that there is a sustained loss of the physiosorbed riboflavin. While this could be problematic for long-duration monitoring (loss of 43% after 54 scans), it nevertheless represents a measurable peak despite the instigation of both repetitive scans and frequent changes in pH (and associated rinses). It could be envisaged that, at least in principle and on the basis of the 54 scans, the 3D-printed NFC-integrated device could potentially fulfill the requirements for scanning over the 72 h duration. Given that the differential period of central line culture versus peripheral culture is typically ≥2 h, it could be presumed that some 12 scans per day are required. [29,30]. In principle, the carbon fiber 3D-printed hub could be viable for 4.5 days—beyond the 3 days recommended for a conventional NFC hub [17,18].

### 3.2. Evaluation of the Printed Catheter Hub with Authentic Infusate

**Scheme 1:**
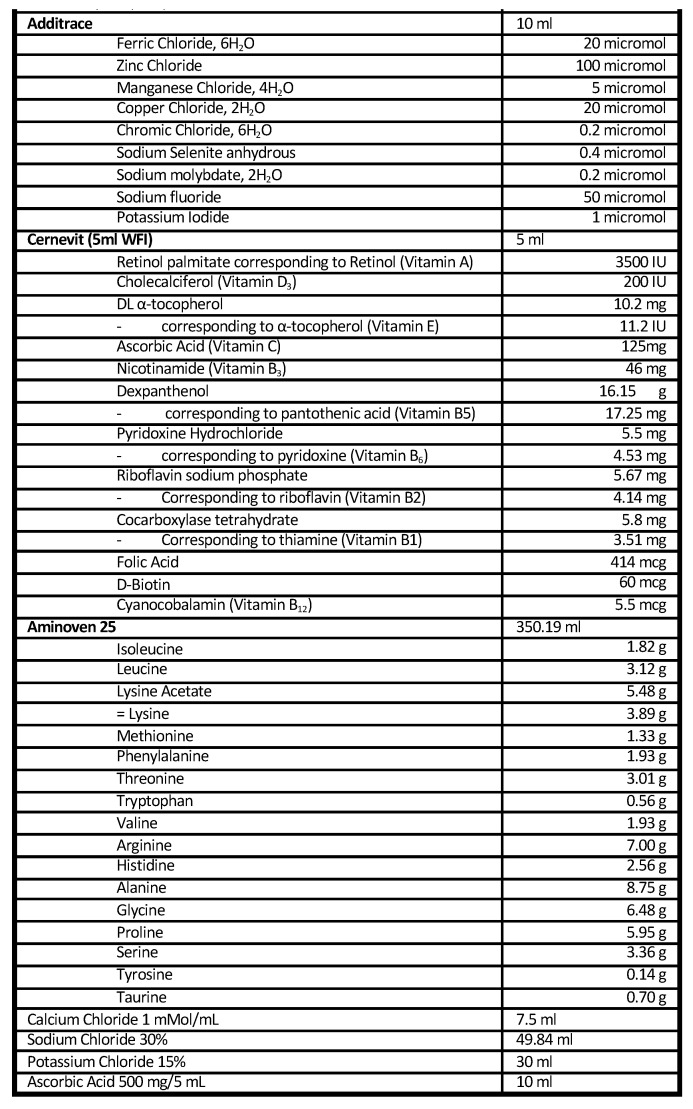
Typical components of the TPN IV infusion solution (Calea UK) used in this study.

Thus far, the electrode medium has been a compositionally simple Britton–Robinson buffer, which offers little challenge to the acquisition of an unambiguous signal. In order to assess the pH-sensitive capabilities of the riboflavin-modified 3D-printed sensor, the system was trialed in total parental nutrition (TPN) intravenous infusions. Numerous risk factors have become associated with catheter line-associated bloodstream infection, including aseptic training, care bundles, catheter duration, and underlying disease. TPN patients are a particular cohort designated as high-risk, and most are treated within community settings. These infusion solutions provide the patient with vital nutrients, but similarly, these same nutrients can also serve as a growth medium for CLABSI-causing microorganisms [31,32,33]. Increased risk was observed in those patients receiving TPN (compared to those who did not). Moreover, Santarpia et al. (2016) observed that, even in the absence of clinical symptoms, some 50% of catheter tips used principally for TPN administration could be categorized as infected [34]. A representative TPN formulation (used for the treatment of small bowel syndrome) is detailed in Scheme 1 and serves to highlight the diversity of components present: carbohydrates, trace minerals, and essential amino acids.

The TPN solution detailed in Scheme 1 contains a variety of potential electroactive interferences (ascorbate, tyrosine, tryptophan, etc.) at appropriate physiological concentrations, which would normally hinder conventional electrochemical sensors. The oxidation of tyrosine and tryptophan can be particularly problematic for electrochemical sensors that employ repetitive/periodic scanning, where their oxidation can lead to the deposition of polymeric material, which can progressively foul the electrode. It is noteworthy that riboflavin’s oxidation peak is observed at more negative potentials (Figure 6A) and, as such, the analytical signal can be obtained within a potential range where the oxidation of the interfering species does not take place, thereby avoiding ambiguous peak profiles (through either competing processes or electrode fouling).

**Figure 8:**
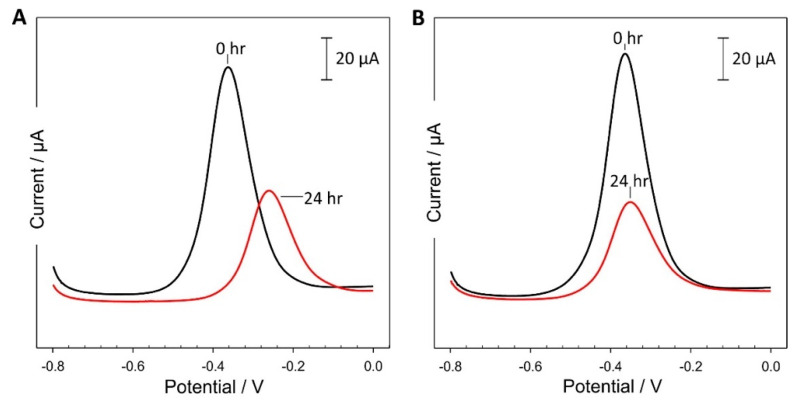
Square wave voltammograms detailing the response of the carbon fiber 3D-printed hub in (**A**) the TPN solution inoculated with Kefir and (**B**) a TPN control without Kefir. Responses were recorded using a two-electrode system with (0.1 M KCl) Ag|AgCl pseudo reference.

In order to simulate bacterial ingress to the line, Kefir grains were used. Kefir is a heterogenous consortium of microbial species that can vary depending on the country of origin; the common, predominant isolates are lactic acid bacteria (i.e., Lactobacillus, Streptococcus species), yeast (i.e., Candida, Saccharomyces species) and acetic acid bacteria (Acetobacter species) [35,36,37,38,39,40,41]. The microbial colony-forming unit (CFU) count during production has an estimated range of 4.6 × 10^3^ to 2.6 × 10^8^ [36,39]—with lactic acid bacteria, yeast, and acetic bacteria concentrations at approximately 10^8^ CFU/g, 10^7^ CFU/g, and 10^5^ CFU/g, respectively [39,40]. It was anticipated that the introduction of Kefir to the TPN solution here would result in a gradual increase in the microbial population, and, as a result, the pH of the medium would fall as fermentation began. Square wave voltammograms detailing the response of the two-electrode carbon fiber sensing hub to TPN before and 24 h after the introduction of Kefir are detailed in Figure 8A. The corresponding control experiment with no Kefir present over the same 24 h period is compared in Figure 8B with a quantitative measure of the changes in pH detailed in Table 1.

Comparing the responses observed in Figure 8, the peak potential was found to significantly change only in the case where kefir grains were present and allowed to ferment throughout the experimental period. The production of lactic and acetic acid resulted in the medium becoming more acidic [39,40,41], and it could be seen that the calculated pH was in close agreement with the commercial pH probe. As mentioned, it is recommended that the NFC component is changed every 72 h [17,18], and, therefore, the proposed sensor system must be capable of enduring periodic scanning over this period. Electrode stability towards repetitive scanning was assessed by cycling the carbon fiber hub system through a series of pH buffers starting from pH 3.07 and incrementing to a maximum of pH 7.96. The square wave voltammogram was recorded in triplicate within each pH BR buffer, and then the entire pH series was repeated three times (in an analogous manner to the experiment summarized in Figure 7). The peak potential was found to drift by 31 mV (equivalent to 0.51 pH units) after a total of 54 scans. This could be attributed to hydroxyl ions attacking the oxidized form of riboflavin that, in turn, showcased a slightly irreversible characteristic. Equally, the instability/degradation of the solid-state pseudo reference electrode could be a factor. It should be noted that a 60% decrease in peak height was seen after repetitive cycling (24 h, Figure 8) with the integrated device in comparison to the 43% decrease found with the more conventional 3 M Ag|AgCl half-cell reference.

### 3.3. Critical Assessment of the Technology and Practice Implications

The stability of the electrode to repetitive scanning was ascertained through consecutive scanning (a total of 54 scans) over a period of 24 h and provided an encouraging assessment of performance. However, scanning over a greater duration is required in order to truly corroborate long-term stability. While the recommended placement of an NFC is 72 h, it is inevitable that hubs are left attached for longer durations and, therefore, more sustained evaluations are required [14,18]. One technical issue that must be highlighted relates to the reference electrode. The system designed here employs a solid-state Ag|AgCl pseudo reference where the potential is determined via the concentration of chloride within the solution present in the catheter lumen. In order to provide an accurate measurement of pH, this requires that the chloride concentration is equal to that used in the calibration solutions. Where there is a discrepancy in chloride concentration in the infusate, the reference potential can change; therefore, the accuracy of the pH is compromised. It is possible that the small error observed here is due to slight variations in chloride concentration between the calibrants and the actual TPN sample. In general, the composition of most intravenous fluids is designed to maintain a constant chloride composition in alignment with the normal blood chloride (0.1 mol/L). An alternative strategy to overcome the dependence on solution chloride may be to employ a solid-state chloride reservoir. This has been performed to good effect through the use of polyvinyl butryal films with wearable sweat sensors [42,43]. The polymer provides an entrapment mesh, incorporating solid potassium or sodium chloride, which provides stable reference potential. It must be noted, however, that such reference systems have been used for discrete sampling, and it is unclear how well they perform under flow regimes.

Training has always been a prime challenge in the management of CVCs—from the point of implantation to the routine day-to-day care of the line. The reliance on human compliance and adherence to the main tenets of aseptic manipulation is problematic and inherently variable [44,45,46,47]. Care bundles were introduced to counter variations in the procedure and to prioritize aseptic techniques, which in many cases has been reported to lead to significant improvements in reducing CLABSI. It must be recognized, however, that improved compliance rates are seldom universal and can be affected by the local environment in which the care is provided. Jeong et al. (2013) revealed that compliance rates were only 37% after the intervention [46], and a more expansive meta-analysis by Ista et al. (2016) highlighted that total compliance is essentially unattainable [48]. The focus has often been placed on the disinfection of the NFC prior to accessing the catheter, and it is surprising that despite the clear hazard posed, it remains a common point of failure [23,30,47,49,50]. The ability to monitor the condition of the hub through the integration of sensors may, therefore, provide a key opportunity to identify these failures and enable interventions that aim for the removal of systemic issues with training and management.

## 4. Conclusions

The carbon fiber mesh electrode, through the adsorption and exploitation of physio-sorbed riboflavin, has been shown to serve as an inexpensive and disposable pH system. The biocompatibility issues of the earlier flavin polymer system have been addressed through the use of riboflavin, as it is already a key vitamin (B2) present within biofluids. Through utilizing the oxidation peak potential of adsorbed riboflavin, an unambiguous signal can be obtained that is free from the overlap caused by the common electroactive interferences typically found in biofluids which could impede conventional voltammetric pH measurements based on quinoid-type redox labels. The carbon fiber 3D-printed hub was evaluated in total parenteral nutrition infusates and was found to be capable of undergoing repetitive scanning, though there was sustained leaching of riboflavin into the solution. It can be envisaged that the modified needle-free connector proposed here could eventually be used as a smart monitor that, in principle, is directly integrated within CVC lines. In general, the strategy outlined should aid in laying a foundation for the further development of a new methodology for detecting changes in bacterial populations through the indirect measurement of pH within the NFC, thereby aiding in the early identification of catheter-related bloodstream infections.

## Figures and Tables

**Table 1:** Peak data obtained from the physiosorbed riboflavin-modified carbon fiber electrode exposed to Kefir-cultured TPN over 24 h and the resulting comparison of calculated pH with that of a commercial pH probe.

With Kefir/h	pH Probe	Average Ep (N = 3)	Calculated pH	% Error
0 h	5.65	−0.363	5.89	4.29
4 h	4.29	−0.259	4.10	−4.33
23 h	3.78	−0.220	3.42	−9.42
24 h	3.74	−0.230	3.60	−3.67

## Data Availability

The generated data and design files are available on request.
